# Reform in Medical Colleges

**Published:** 1876-07

**Authors:** 


### EDITORIAL, ETC.

---

*Reform in Medical Colleges.*—From the proceedings of the Association of the Representatives of American Medical Colleges, held in Philadelphia, June 2nd and 3rd, 1876, we take the following: it being

*Resolved,* That the action of the convention shall not be considered binding upon the colleges represented unless endorsed by their respective faculties.

*Whereas,* The practice of reducing or remitting in individual cases the established fees of a college has the objectionable feature of discriminating between students who may be equally deserving, and opening the door to possible gross abuses: therefore

*Resolved,* That this convention regards the above privilege as one to be deprecated in general, and, if put into practice at all, to be exercised both rarely and reluctantly, and only in unusual circumstances, and after unsolicited application by proven deserving candidates.

That anything like a wholesale system of such reduction or remission of established fees, or any open solicitation of recipi ents of such favors be regarded as in the highest degree improper, and that any college indulging in such practices deserves to forfeit its place on the *ad eundem* list of medical colleges.

That it is the opinion of this convention that no two consecutive sets of lecture tickets shall be regarded as fulfilling the usual pre-requisites of instruction for graduation, where the time between the beginning of the first course and the end of the second is less than fifteen months.

That no medical faculty should issue a diploma not bearing the graduate's name.

That it is the sense of the convention that the diploma fee should not be abolished.

*Whereas,* A knowledge of the elementary branches of medicine should precede a study of the practical branches:

*Resolved,* That, in the hope of inducing students to prolong and systematize their studies, this convention recommends to all medical colleges to offer to students the option of three courses of lectures, after a plan similar to the following . Students who have attended two full courses of lectures on anatomy, chemistry, materia medica and physiology, may be examined upon any of these subjects at the end of their second course. During their third course such students may devote themselves to the lectures upon the theory and practice of medicine, surgery, obstetrics and diseases of women and children, upon which subjects only they shall be examined at the final examination for the degree of M. D.—their standing, however, to be determined by the results of both examinations.

*Resolved,* That in the opinion of this Association, medical colleges ought not to recognize or hold fellowship with any school or its alumni in which irregular medicine is taught as a part of the curriculum.

No degree in medicine should be conferred, under any circumstances, except after an examination in person of the candidate upon all the branches of medicine.

